# Mucins in ovarian cancer diagnosis and therapy

**DOI:** 10.1186/1757-2215-2-21

**Published:** 2009-12-24

**Authors:** Subhash C Chauhan, Deepak Kumar, Meena Jaggi

**Affiliations:** 1 Cancer Biology Research Center, Sanford Research/USD, Sioux Falls, SD, USA; 2 Department of OB/GYN and Basic Biomedical Science Division, Sanford School of Medicine, Sioux Falls, SD, USA; 3 Department of Biological and Environmental Sciences, University of the District of Columbia, Washington, DC, USA

## Abstract

Ovarian cancer is the most lethal gynecologic malignancy and the five-year survival rate is only 35% after diagnosis. Epithelial ovarian cancer is a highly metastatic disease characterized by widespread peritoneal dissemination and ascites. The death incidences from ovarian cancer could be significantly lowered by developing new methods for the early diagnosis and treatment of this fatal disease. Several potential markers have been identified recently. However, mucins are the most promising markers for ovarian cancer diagnosis. Mucins are large extracellular, heavily glycosylated proteins and their aberrant expression has been implicated in the pathogenesis of a variety of cancers, including ovarian cancer. This review will summarize known facts about the pathological and molecular characteristics of ovarian cancer, the current status of ovarian cancer markers, as well as general information about mucins, the putative role of mucins in the progression of ovarian cancer and their potential use for the early diagnosis and treatment of this disease.

## Ovarian Cancer

The life-time risk of having ovarian cancer is 1 in 70 women. This is the fifth leading cause of death for women in developing countries [1,2]. According to epidemiological studies, age is a common risk factor of ovarian cancer because the ovaries of post-menopausal women become smaller and folded. This folding results in deep cleft formations and formation of smaller cysts lined with ovarian surface epithelial (OSE) cells [3-6]. The other risk factors are: nulliparity, family history, history of fertility drug use and endocrine disorders. Multiparity, use of oral contraceptives, pregnancy and lactation all are associated with lower risk of ovarian cancer because of the decreased number of ovulation cycles [6-10]. Molecular alterations are also known to occur in ovarian cancer. These molecular alterations include mutation in the p53 gene which is known to be involved in DNA damage repair. Mutation in BRCA1 and BRCA2 has also been reported in ovarian tumors [11-15]. Inactivation or downregulation of tumor suppressor genes and amplification of oncogenes is also a potential cause of ovarian cancer. In ovarian tumors, the downregulation of OVCA1 and OVCA2 (tumor suppressor genes present in normal ovary) is reported, while their functions in normal ovary are not well known [11,16]. In contrast, overexpression/amplification of certain oncogenes like C-MYC, RAS, AKT, EGFR (ErbB1 or HER1), HER2/neu (ErbB2), CSF1 C-MYC, etc., is also well known in ovarian tumors [3-5,11,14,17-20].

## Ovarian Cancer Staging and Histological Types

Phenotypically, the following types of epithelial ovarian cancers (90%) are classified based on their expressed properties related to the epithelium of the fallopian tube (serous tumors), proliferative endometrium (endometroid), endocervix or colonic epithelium (mucinous tumors), gestational endometrium (clear cell carcinoma), or the urinogenital tract (transitional or Brenner tumors) (Table 1). The remaining 10% of ovarian tumors are gonadal-stromal tumors (6%), germ cell tumors (3%) and metastatic tumors (1%) [5] (Table 1). The histological classification of ovarian tumors suggests four different stages in ovarian cancer: stage I (tumors involve one or both the ovaries, 5 year survival 60-90%), stage II (tumors involve one or both ovaries with pelvic extension, 5 year survival 37-66%), stage III (tumors involve one or both ovaries with intraperitoneal metastasis outside the pelvis, retroperitoneal nodes or both, 5 year survival 5-50%) and stage IV (tumors involve one or both ovaries with distant metastases, i.e. to lungs or liver, 5 year survival 0-17%) [5,21] (Table 2). The majority (90%) of ovarian cancers are epithelial ovarian carcinomas (EOC) which are thought to arise from the ovarian surface epithelium (OSE). OSE is the outermost mesothelial (peritoneal) lining and least component of the normal ovary, with no unique feature or known major functions. In addition, the early changes and minor anomalies remain undetected in this tissue [3,5,20]. Due to the anatomic location and the lack of early symptoms, it has become a difficult task to differentiate normal OSE, metaplasia, benign epithelial tumors and borderline tumors. Ovarian cancer can be treated effectively if detected at an early stage; but unfortunately, at the present time most of the ovarian tumors are not diagnosed before an advanced stage (stage III and IV) primarily due to the lack of reliable biomarkers of early diagnosis. Since most ovarian cancers are of epithelial nature and mucins are considered to be the hallmark of epithelial cells, the expression profile of mucins may serve as a potential diagnostic/prognostic and therapeutic target. In this article, we have compiled available information on the expression profile of different mucins in ovarian tumors and their potential role in ovarian cancer diagnosis and treatment.

**Table 1:** Classification of ovarian tumors

**Epithelial ovarian tumors (90%) Mostly diagnosed after the age of 50**.	Germ cell neoplasm (3%) Mostly diagnosed under the age of 30	Gonado-stromal tumors (6%) No particular pattern with age
Serous	Teratomas	Granulosa cell tumors
Mucinous	Mature cyst teratomas	Thecomas
Endometroid	Immature teratomas	Fibrosarcomas
Clear cell	Dysgerminomas	Sertoli cell tumors
Transitional cell or Brenner tumors	Yolk sac tumors Embryonal carcinomas	Leydig cell tumors

Metastatic tumors: Ovaries may have tumors due to secondary metastatis of stomach, colon, pancreas, appendix, breast, and hematopoietic system.

**Table 2:** Stage and Features of the Ovarian Tumors

Stage	Features	% 5 year Survival
Stage I	Tumor growth is limited to the one or both the ovaries	60-90

Stage II	Tumor growth in the one or both the ovaries with extension in the pelvis	37-66

Stage III	Tumor growth involves one or both ovaries with extension and intraperitoneal metastasis extended to the bowel, to the lining of the abdominal cavity, or to the lymph nodes	5-50

Stage IV	Tumor growth in one or both ovaries with distant metastases to other organs such as lungs liver or in the chest	0-17

## Mucins

Being that 90% of ovarian cancers are of epithelial origin, mucins may be attractive candidates for the detection of early stage ovarian cancer [1,2,5]. Mucins, large extracellular proteins, are heavily glycosylated with oligosaccharides and are generally known for providing protection to the epithelial tissues under normal physiological conditions [22-24]. Mucins are usually secreted by the epithelial tissues which remain in contact with relatively harsh environments such as airway epithelium, stomach epithelia, epithelial lining of intestine and ductal epithelial tissue of liver, pancreas, gall bladder, salivary gland, lachrymal gland, etc. In these tissues, epithelial cells are exposed to a variety of microorganisms, toxins, proteases, lipases, glycosidases and diverse microenvironment fluctuations that includes pH, ionic concentration, oxygenation, etc. [22-25]. All mucins share general characteristics. For example, they have repetitive domains of peptides rich in serine, threonine, and proline in their backbone. Serine and threonine are sites for O- and N-glycosylation. Presence of the tandem repeat domain which varies in number, length and O-glycosylation is the common structural feature of all mucins [23,26-29]. Their general structure and biochemical composition provides protection for the cell surface and specific molecular structures regulate the local microenvironment near the cell surface. In addition, mucins also communicate the information of the external environment to the epithelial cells via cellular signaling through membrane-anchored mucins [22-24,29]. It appears that mucins have the capability of serving as cell surface receptors and sensors and conducting signals in response to external stimuli for a variety of cellular responses like cell proliferation, cell growth, differentiation and apoptosis. These reports suggest that the aberrant expression of mucins may be implicated in the development and progression of ovarian cancer.

## Type of Mucins

Currently, there are twenty known mucins which have been placed in two categories: secreted mucins (gel forming: MUC2 [30], MUC5AC [31], MUC5B [32], MUC6 [33], and non-gel forming: MUC7 [34] MUC8 [35] and MUC11[36]), and membrane bound mucins (MUC1[26], MUC3 [37], MUC4 [38], MUC9 [39], MUC10 [40], MUC12 [36], MUC13 [41], MUC16 [42,43], MUC17 [44], MUC18 [45] and MUC20 [46]).

## Mucin Expression in Normal Ovary and Nonmalignant Ovarian Cell Lines

Goblet cells or glandular structures are not present in normal ovaries and, therefore, the normal ovarian tissues are not expected to express secretory mucins. Ovarian surface epithelium (OSE) expresses a mixed epithelo-mesenchymal phenotype and is the only compartment known to express mucins. MUC1 is the only well known mucin which is expressed by the OSE at a detectable level [3,4]. Cultured nonmalignant ovarian epithelial cell lines also express MUC1 (a membrane associated mucin) and MUC5AC (a secreted mucin) [47].

## Mucin Expression in Ovarian Tumors

**Figure 1:**
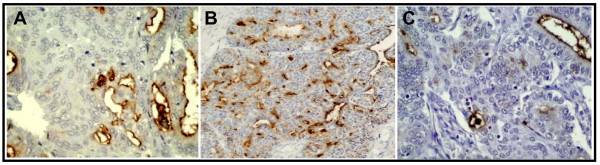
**Expression of MUC1 (A), MUC13 (B) and MUC16/CA125 (C) trans-membrane mucins in ovarian tumors**.

The expression of mucin genes by ovarian epithelial cells has not been studied in detail and only a few reports are available to address this issue. Phenotypically, EOCs are among the most variable tumors of any organ in that they may express ovarian tumor cells structurally related to the epithelium of different organs [4]. It has been shown that malignant ovarian tumors often express more mucins than benign and borderline ovarian tumors. Different studies (Table 3) on the expression of mucins in ovarian tumors have shown overexpression of MUC1, MUC2, MUC3, MUC4, MUC5AC and MUC16 or CA125 [4,47-51]. In agreement with these studies, we also observed overexpression of MUC1, MUC4 and MUC16 in several ovarian tumors [52] with no or an undetectable level of MUC4 and MUC16 in normal ovarian tissues. In northern blot analysis a higher expression of MUC3 and MUC4 was reported in early stage ovarian tumor samples compared to the late stage ovarian tumor samples and it was proposed that they provided a protective function in ovarian cancer [47]. However, in our study we did not see this correlation with MUC4 [52]. The overexpression of MUC1 in various types and stages of ovarian tumor samples is reported in several studies [47,49,50]. Recently, our laboratory has identified aberrant expression of a novel membrane anchored mucin, MUC13, in ovarian cancer. In this study, MUC13 expression was undetectable in normal and benign ovarian samples while 66% of epithelial ovarian cancer samples showed a significantly higher MUC13 expression. MUC13 was predominantly localized on the apical membrane and in the cytoplasm. Moreover, MUC13 expression was significantly (p < 0.05) higher in mucinous and Brenners type of samples compared to other histological types of ovarian cancer samples and adjacent normal ovary samples [53]. The expression pattern of certain membrane bound mucins in ovarian tumors is shown in Figure 1.

**Table 3:** Comparative expression profile of mucins in different stages and histological types of ovarian cancer

Gene	Normal Ovary	Borderline (Mucinous)	Low Stage (Stage 1-2)	High Stage (Stage 3-4)	Detection method
MUC1	+/-	++	+ to +++ (in all histological types i.e. C, M, E, S)	+ to +++ (in all histological types i.e. C, M, E, S)	ISH, NB, IHC [47-50]
MUC2	ND	+++	+++ (all histological types, primarily in mucinous type)	+ to ++	ISH, NB, IHC [47-51]
MUC3	ND	+++ (primarily in intestinal phenotype	+++ (E, M)	- to +	ISH, NB [47,48]
MUC4	-	+++ (primarily in endocervical phenotype)	+++ (all types i.e. C, M, E, S)	- to ++	ISH, NB, IHC [47,48]
MUC5AC	ND	++ (primarily in gastric surface cell or mucinous type)	++ (E, M, S)	++	ISH, NB, [47,48]
MUC5B	ND	++ (Express primarily in endocervical phenotype)	++ (C, S)	- to +	ISH, NB [47,48]
MUC13	ND	+	+++ (S, M)	++ (S, M)	OMA, TMA, IHC [53,97]
CA125/MUC16	-	- (express in non-mucinous borderline tumors	- to +++ (rarely express in mucinous tumors)	+ to +++ (rarely express in mucinous tumors)	IHC [76-79]
MUC17	-	+	-	-	[44,97]

Note: C, M, E, and S are abbreviated for clear cell, mucinous, endometroid and serous histological types of ovarian tumors, respectively.

ISH, in-situ hybridization; NB, northern blotting; IHC, immunohistochemistry, TMA, tissue microarray, OMA, oligonucleotide microarray

## Pathological Roles of Mucins in Ovarian Cancer

The acquirement of an invasive phenotype is one of the pivotal features of malignant ovarian cells. In order to progress and metastasize, ovarian cancer cells must lose cell contacts with neighboring cells, traverse the basement membrane and migrate through stroma to reach blood vessels or the lymphatic system. Mucins may be implicated in the exfoliation, dissemination and invasion of the ovarian cancer cells due to the highly glycosylated extracellular domain, which may protrude up to 200-2000 nm above the cell surface [54-56]. The overexpression of mucins can effectively interfere with the function of cell adhesion molecules by steric blocking of the interaction of the cell surface molecules. MUC1 is known to suppress cell aggression and cell adhesion properties by interfering with the functions of E-cadherin and other cell adhesion molecules in MUC1 overexpressing breast cancer cells [54-56]. In addition to this, mucins may also be involved in the invasion of the basement membrane by modulating cell-matrix attachment because of their diffused and basal localization in tumor cells. Mucins may also have an immunosuppressive effect by covering the surface of tumor cells and enabling access to the immune responsive cells [24,54-60]. The juxtamembrane domain of the membrane-bound mucins is known to promote cell proliferation by intercellular signaling mediated *via *one of their two/three EGF-like domains [24,55-61]. Moreover, the cytoplasmic tail of mucins like MUC1 is known to induce several cell signaling pathways, which promote the cell growth and proliferation in a variety of cancer cells [24,55-57,61-64]. Additionally, our recent study demonstrates that exogenous MUC13 expression induced morphological changes, including scattering of cells. These changes were abrogated through c-jun NH2-terminal kinase (JNK) chemical inhibitor (SP600125) or JNK2 siRNA. Moreover, a marked reduction in cell-cell adhesion and significant (p < 0.05) increases in cell motility, proliferation and tumorigenesis in a xenograft mouse model system were observed upon exogenous MUC13 expression. These cellular characteristics were correlated with up-regulation of HER2, p21-activated kinase1 (PAK1) and p38 protein expression [53]. Additionally, recent studies have shown the role of MUC16/CA125 in ovarian cancer metastasis. MUC16 mucin interacts with the glycosylphosphatidylinositol anchored glycoprotein mesothelin at high affinity and facilitates the peritoneal metastasis of ovarian cancer cells [65,66]. Moreover, MUC16/CA125 expression has been shown to inhibit the cytotoxic responses of human natural killer (NK) cells and downregulate CD16 activity in ovarian cancer cells. It has also been shown that MUC16/CA125 selectively binds to 30-40% of CD16^+ ^NK cells in EOC patients. These studies suggest immunosuppressive properties of MUC16/CA125 [67]. These above mentioned findings demonstrate the aberrant expression of mucins in ovarian cancer and show that mucin expression may alter the cellular characteristics of ovarian cancer cells and also imply a significant role of mucins in the pathogenesis of ovarian cancer.

## Mucins as Serum Marker of Ovarian Cancer

The structural characteristics of mucins suggest the presence of potential proteolytic cleavage sites in most mucin genes and several are known to cleave at the cell surface. Mucins, which are normally confined to the epithelial surfaces, become exposed to circulation and their overexpression may establish their potential as a tumor marker and/or diseased condition. Mucins already have shown their great potential as serum markers of ovarian and various other tumors. Aberrant O-glycosylation of mucins is particularly prominent in epithelial cancers. This feature has been termed "glycodynamics". These heterogeneously O-glycosylated mucins aberrantly enter the bloodstream in malignant conditions which provide diagnostic biomarkers for detection and monitoring of cancer. Although mucins are rapidly degraded by glycan-recognizing hepatic clearance receptors in the liver, small subsets of carcinoma mucins remained unrecognized by clearance systems. Thus, circulating cancer mucins used as clinical diagnostic markers likely represent only the clearance-resistant "tip of the iceberg" [68]. For example, O-glycans on circulating MUC16 recognized by antibody CA125 provide for diagnosis and monitoring of ovarian cancers [42]. CA125, an established serum marker of ovarian tumors, has been recently identified as a member of a mucin family and named MUC16 [42,43,69]. MUC16 is a large, heavily glycosylated transmembrane mucin. Several studies have shown the importance of CA125/MUC16 in ovarian cancer diagnosis. In fact, an elevated level of CA125/MUC16 is a gold standard non-invasive test for ovarian cancer diagnosis [70,71]. A decrease in CA125 can provide a surrogate marker to determine the response to chemotherapeutic drug(s) during the treatment procedure [72]. Moreover, antigens such as CA19-9, CA50, and CA242 are also the serum markers of various malignant conditions and are present on heavily glycosylated, high molecular weight mucins [22,73,74]. In breast cancers, serum MUC1 measured by CA15-3 is a well established assay and has been shown to correlate with the clinical course [75]. MUC1 and MUC4 are also known to be overexpressed in ovarian tumors. Despite having a great importance in ovarian cancer, CA125 does not display an elevated serum level in over 50% of the women with early stage tumors because this antigen is not expressed in most early stage ovarian tumors [1,76-79]. Additionally, an elevated level of CA125 was observed in some other (pancreatic, breast, liver, bladder and lung) cancers, benign conditions (diverticulitis, uterine fibroids, endometriosis, and ectopic pregnancy) and physiological conditions (pregnancy and menstruation). Therefore, the discovery of new serum tumor markers capable of complementing CA125 may allow for the development of a reliable test for the early stage diagnosis of ovarian cancer. Our recent and some previous studies showed the overexpression of MUC4 in a majority of early stage ovarian tumors and a combined panel of MUC1, MUC4 and MUC16 dramatically increased the sensitivity of MUC16 staining test [52]. Additionally, a recent study suggests the overexpression of MUC4 in ovarian carcinoma cells present in peritoneal effusions [80]. Furthermore, our laboratory has recently identified the aberrant expression of a novel transmembrane mucin, MUC13, in ovarian tumor samples compared to normal/benign ovarian tissue samples [53]. Like other membrane-associated mucins, MUC4 and MUC13 also have a proteolytic cleavage site in its structure which may allow the cleavage of the extracellular part of MUC4 and MUC13 and their release in the blood stream [29]. A similar process occurs in case of MUC1 and MUC16. These data suggest that a combined panel of different mucins may improve sensitivity and accuracy of the currently used serum based diagnosis of ovarian cancer. Further, aberrant mucin expression may be immunogenic and may elicit a potent antibody response. This antibody response may also serve as a disease indicator. A recent study demonstrated the presence of MUC1 antibodies in blood plasma samples which was inversely correlated with risk of ovarian cancer [81]. These studies suggest that the aberrant expression of mucins holds great promise to serve as a surrogate marker of ovarian cancer and ovarian cancer prognosis.

## Use of Mucins in Radioimmunodiagnosis (RID) and Radioimmunotherapy (RIT)

Monoclonal antibodies against mucins may have potential applications in improving the diagnosis and therapy of ovarian tumors, although very few published studies are available to address this issue, so far, and continued investigations are certainly required. The much higher expression of mucins (MUC1, MUC4, MUC5AC, MUC13 and MUC16) in ovarian tumors compared to the surrounding normal tissues can be exploited for the purpose of radioimmunodiagnosis (RID) and radioimmunotherapy (RIT). MUC1 monoclonal antibodies radiolabeled with γ-emitting radioisotopes like ^99m^TC and ^111^In have been successfully used for the radioimmunodiagnosis of various malignancies [82]. As an extension of this technique, monoclonal antibodies to the mucins, radiolabeled with β-emitting isotopes such as ^67^Cu, or ^188^Re, may be employed for the irradiation of spreading tumor cells (radioimmunotherapy) while sparing normal cells [82-84]. At present, MUC1 and MUC16 are the best and only characterized mucins and monoclonal antibodies against MUC1 and MUC16 are under preclinical and clinical investigations for ovarian cancer treatment (Table 4). Therapeutic efficacy of anti-MUC1 MAb (HMFG1: anti-human milk fat globules) radiolabeled with ^90^Y, ^186^Re and ^131^I was investigated in an OVCAR3 ovarian cancer xenograft model. These radiopharmaceuticals significantly improved survival in treated mice compared to control mice. Similarly, radiolabeled MUC16 MAbs also caused significant delay in animal death. MUC13 is another potential mucin which is highly expressed on the surface of ovarian cancer cells, indicating its potential as a target for RID and RIT. An emerging concept in radioimmunotherapy is nano-radioimmunotherapy (Nano-RIT). In these studies radiolabeled antibodies are coupled with drug loaded liposomes or nanoparticles. This approach will overcome some of the major obstacles associated with conventional strategies and will improve tumor uptake and retention time of radioimmunoconjugates [85,86]. The radioimmunoconjugates can be safely administered *via *an intravenous route despite the fact they are mouse monoclonal antibodies and capable of inducing human anti-mouse antibody (HAMA) responses. However, this problem can be minimized in the future by using modern antibody engineering techniques [87].

**Table 4:** Some mucin-based and other emerging therapies for ovarian cancer treatment [88-94]

Antibody targeting	Vaccines		
	
	Antibody-based	Antigen-based	Cell-based
Anti-HER2/neu antibody (Herceptin) [In use]	Idiotypic vaccination with anti-MUC1 HMFG1MAb [Phase I trial]	MUC1 presenting Immunogens [Phase I]	Fusions of ovarian carcinoma cells and dendritic cells (DC) [Preclinical]

^90^Y-labelled anti-MUC1 HMFG1 MAb [Phase 1]	Anti-CA-125 B43.13 MAb vaccine (OvaRex) [Phase IIb]	Peptides derived from a folate binding protein [Phase 1]	MUC1 RNA transfected dendritic cells [Preclinical]

^131^I-labelled OC125 MAb [Phase I/II]	Anti-idiotypic antibody ACA-125 vaccine [Phase I/II]	Synthetic Lewis (y)-protein conjugate vaccine [Phase 1]	Genetically engineered GM-CSF producing tumor cells

^131^I-labelled MOv8 chimeric MAb [Phase 1]		Her2/neu presenting peptides vaccines [Phase 1]	Her2/neu and MUC1 peptide pulsed dendritic cells [Pilot study]

Nano-RIT with CA125 and anti-HER2 MAb [Under investigation]		Theratope STn-KLH cancer vaccine [Phase 1]	Dendritic cells pulsed with tumor-lysate

## Anti-Cancer Vaccines Based on Mucins

In recent years, projects associated with the development of tumor vaccines have received considerable attention (Table 4). A further possible approach involves the use of mucins as a vaccine and target for immune responses (Table 4) [88,89]. Three types of strategies can be employed for vaccine development: antibody-based, antigen-based and cell-based. As we mentioned earlier, certain membrane anchored mucins which are over/aberrantly expressed in ovarian cancer can be targeted for monoclonal antibody generation and anti-cancer vaccine development. Antibody generated against a tumor antigen can trigger potent antibody-dependent cellular cytotoxicity and T-cell response. Additionally, monoclonal antibodies can persuade anti-idiotypic antibodies that mimic the epitopes in tumor antigens and can elicit a potent anti-cancer response in patients. For an anti-cancer vaccine, synthetic peptide or DNA that encodes for a tumor antigen can be administered to the patient and over time the patient will develop an immune response by activation of cytotoxic T cells. In a cell-based vaccine approach, tumor cells of the same patient (autologous) or a different patient (allogeneic) or dendritic cells (activated by cancer antigen) are administered to the cancer patient to stimulate the immune system. The induction of potential anti-MUC responses may provide potential benefits in targeting tumors overexpressing mucin antigens. MUC1 has been successfully used as a target for immuno-directed therapies and as a marker of disease progression [88-90]. The efficacy of the immune response to mucins or mucin peptides can be effectively augmented by conjugation of immune adjuvant and/or carrier proteins like Bacille Calmette-Guerin (BCG) and keyhole limpet hemocyanin (KLH). A cognate of the MUC1 peptide conjugated with KLH and *Quillaja saponaria *(QS-21) has entered into clinical trials for prostate cancer [91,92]. The use of naked DNA is another attractive and relatively simple approach for vaccination studies. MUC1 cDNA has been used as a cancer vaccine in mouse models and has been shown to result in long-term growth suppression of tumors [93,94]. Additionally, dendritic cells pulsed with mucin derived peptides were able to induce a potent cytotoxic T-cell response and provide therapeutic benefits [95,96]. For ovarian tumors, which are known to overexpress mucins, this may be a potential treatment approach with a better survival outcome.

## Conclusions

The mucin gene family has considerable potential importance in the cell biology, diagnosis and treatment of ovarian malignancies. Various studies have shown the overexpression of MUC1, MUC2, MUC3, MUC4, MUC5AC and MUC16 in a variety of ovarian tumors. In particular, a combined panel of MUC4, MUC5AC, and MUC16 may offer an effective and reliable diagnostic system and target for the management of various histological grades and types of ovarian cancer, although their biological functions are not clearly defined. The development of new molecular biology techniques will allow researchers to determine the biological role of mucins in the process of ovarian tumor progression and response to therapy. The gene locus of the majority of mucin genes has been identified and, therefore, may be a potential target for future gene-based therapies, including immunoliposome targeted techniques. The use of mucins as targets for radioimmunodiagnosis and radioimmunotherapy is also being explored and appears to be a potential approach for the diagnosis and treatment of ovarian tumors which overexpress mucins. The advancement in the area of antibody engineering techniques provides an opportunity to produce single-chain, divalent, tetravalent and humanized antibody constructs from murine monoclonal antibodies. These molecules will be significantly less immunogenic to the human host than their intact mouse Ig counterparts, and may allow repeated intravenous/intraperitoneal administrations of targeting radioconjugated molecules, improved tumor tissue penetration due to reduced physical size with a minimal or no risk of an HAMA response. In the light of available information, we conclude that switching of mucin genes occurs in ovarian cancer, which can be utilized for the early diagnosis and treatment of ovarian tumors.

## Competing interests

The authors declare that they have no competing interests.

## Authors' contributions

SCC drafted the manuscript. DK and MJ participated in substantial contribution to revising of the manuscript. All authors read and approved the final manuscript.
